# Asking for a Commitment: Violations during the 2007 Match and the Effect on Applicant Rank Lists

**DOI:** 10.5811/westjem.2015.1.24462

**Published:** 2015-02-25

**Authors:** H. Gene Hern, Brian Johnson, Harrison J. Alter, Charlotte P. Wills, Eric R. Snoey, Barry C. Simon

**Affiliations:** Alameda Health System - Highland Hospital, Department of Emergency Medicine, Oakland, California

## Abstract

**Introduction:**

Applicants to residency face a number of difficult questions during the interview process, one of which is when a program asks for a commitment to rank the program highly. The regulations governing the National Resident Matching Program (NRMP) match explicitly forbid any residency programs asking for a commitment.

**Methods:**

We conducted a cross-sectional survey of applicants from U.S. medical schools to five specialties during the 2006–2007 interview season using the Electronic Residency Application Service of the Association of American Medical Colleges. Applicants were asked to recall being asked to provide any sort of commitment (verbal or otherwise) to rank a program highly. Surveys were sent after rank lists were submitted, but before match day. We analyzed data using descriptive statistics and logistic regression.

**Results:**

There were 7,028 unique responses out of 11,983 surveys sent for a response rate of 58.6%. Of those who identified their specialty (emergency medicine, internal medicine, obstetrics and gynecology [OBGYN], general surgery and orthopedics), there were 6,303 unique responders. Overall 19.6% (1380/7028) of all respondents were asked to commit to a program. Orthopedics had the highest overall prevalence at 28.9% (372/474), followed by OBGYN (23.7%; 180/759), general surgery (21.7%; 190/876), internal medicine (18.3%; 601/3278), and finally, emergency medicine (15.4%; 141/916). Of those responding, 38.4% stated such questions made them less likely to rank the program.

**Conclusion:**

Applicants to residencies are being asked questions expressly forbidden by the NRMP. Among the five specialties surveyed, orthopedics and OBGYN had the highest incidence of this violation. Asking for a commitment makes applicants less likely to rank a program highly.

## INTRODUCTION

The residency interview process creates much anxiety among senior medical students and residency programs each year. The National Resident Matching Program (NRMP) was created in 1952, to help medical students navigate the process and in part mitigate some of the high-pressure tactics of recruiting that were common in that era. Prior to the NRMP, students often were forced to accept appointments as early as the second year of medical school, even prior to any clinical clerkship.^1^ The NRMP allowed applicants to make decisions on a uniform schedule, ostensibly without pressure from residency programs. At the center of the NRMP was the Match Participation Agreement (MPA), which outlined restrictions on persuasion.^2^ Per the MPA, applicants and programs may not solicit either a verbal or written commitment or suggest that ranking is contingent upon a commitment. However, anecdotal accounts and published studies suggest programs violate this rule. In 1998, senior medical students from three medical schools reported a 34% rate of inappropriate commitment requests from programs.^3^ Emergency medicine applicants in 2005 and 2006 reported an 8% rate though the survey response rate was 28%.^4^

The current literature suggests that both applicants and residency program directors feel that the application process retains inherent dishonesty. Applicants feel uncomfortable and forced to lie about ranking a program while program directors distrust the information they receive from applicants.^3^,^5^–^7^ When multiple programs request a commitment – either overt or subtle – the applicant may have less incentive to report an individual program, considering commitment requests commonplace.^7^ We performed a national, multi-specialty survey to describe the prevalence and burden of commitment violations during the 2006–2007 interview season.

## METHODS

We surveyed applicants in the 2006–2007 NRMP in five specialties: emergency medicine, internal medicine, general surgery, obstetrics and gynecology (OBGYN), and orthopedic Surgery on various topics as part of a larger project understanding the residency match milieu.^8^ We solicited information from residency match subjects on NRMP match rule violations.

The survey instrument was developed in conjunction with a survey design and protocol specialist at UC Berkeley, School of Public Health. The instrument was piloted among residents (a population close in age and experience to our survey population) and faculty for clarity. The pilot and revisions were in accordance with survey design methodology to maximize validity and reliability.

We included both categorical and preliminary applicants for medicine and surgery specialties; however, we collapsed them into their respective specialty for analysis. Study participants were recruited through an electronic mail message sent by the Electronic Residency Application Service (ERAS) in February of 2007. ERAS is a centralized service that transmits medical student residency applications to residency program directors. ERAS referred the residency applicants to a public instrument for study enrollment. The instrument was located on a public website (www.surveymonkey.com) and all data remained anonymous with a custom ID generated by ERAS and securely encrypted. Study participants were informed on the survey that the information would remain confidential and only study investigators would have access to their anonymous responses. The electronic message was sent twice (one week apart) after residency rank lists were submitted by applicants and programs but before match results were known. All data analyzed were received within two weeks of the initial email. We discarded data received after match results were public.

Applicants were asked to recall if, during the course of their interviews, they were asked to provide a commitment, verbal or otherwise, that they would rank a program highly. The survey also asked what effect such questions had on their decisions to rank that program. Respondents were asked to characterize how many programs solicited a commitment. The survey also collected demographic information including age, gender, marital status, number of children, race/ethnicity and religious affiliation. Participants also described the specialty they were applying to, number of interviews offered, and number of interviews completed by the applicant.

Surveys were imported into a Microsoft Excel spreadsheet (Redmond WA). We calculated frequencies for all survey variables. The primary outcome was solicitation of commitment by a residency program. Commitment request was stratified by the covariate variables. We calculated differences using chi square analysis for categorical variables and t-test for univariate variables. Statistical analysis was completed using both STATA (Version 10.1, STATA Corporation, College Station, Texas ) and SAS (Version 9.2, Cary NC).

The Institutional Review Board at our home institution approved the instrument and protocol prior to distribution.

## RESULTS

### Overall Prevalence

Questionnaires were sent to 11,983 applicants and 7,028 replied, resulting in a response rate of 58.6%. Overall, 1,380 or 19.6% of applicants (1380/7028) were asked for a commitment (verbal or otherwise) to rank a program highly. Of those who were asked for a commitment, 540 (39.1%) were asked by one program, 333 (24.1%) by two programs, 187 (13.6%) by three, and 112 (8.1%) by four.

### Specialty and Demographic Differences

Among those respondents who indicated their specialty, orthopedics had the highest overall prevalence at 28.9% (372/474) followed by OBGYN (23.7%; 180/759), general surgery (21.7%; 190/876), internal medicine (18.3%; 601/3278), and finally, emergency medicine (15.4%; 141/916) (Table 1).

Women were more likely to be asked for a commitment. Almost 23% of women (732/3194; 22.9%) were asked for a commitment while only 18.2% of men (612/3358) were solicited. This difference was shown to be statistically significant with p<0.001. There was no statistically significant difference in proportion of applicants asked for commitment by age, race/ethnicity, marital status, number of children, and religious status.

### Comfort Level and Rank List Effect

Of the applicants asked to provide a commitment, 1,030 (74.6%) felt either uncomfortable or very uncomfortable sharing this information (Table 2). Furthermore, applicants solicited for a commitment were less likely to rank the program. Among applicants approached for committal, 388 of 1,380 (28.1%) stated they were ‘less likely’ to rank the program and 142 of 1,380 (10.3%) were ‘much less likely’ to rank the program. Fewer than half the respondents said commitment solicitation had no effect on their decision to rank the program (46.9%; 647/1380) (Table 3).

Among those applicants asked for a commitment, OBGYN applicants felt the most uncomfortable – 142 of 179 (79.3%) respondents were either somewhat or very uncomfortable sharing this information. This was followed by orthopedics at 75.5% (102/135), emergency medicine 75% (105/140), internal medicine 74.5% (445/597), and surgery 70.7% (133/188) (Table 4).

When asked if questions of commitment affected their ultimate rank lists, emergency medicine residents stated it would affect them the most. Almost half of emergency medicine residents were less likely to rank a program based on this question (44.6%; 62/139). Lower percentages were given by applicants in OBGYN (42.5%; 76/179), internal medicine (39.4%; 234/594), orthopedics (33.6%; 45/134), and surgery (32.8%; 62/189) (Table 5).

## DISCUSSION

Results of our national survey suggest that residency applicants routinely experience questions of residency commitment. Almost one in five respondents in the surveyed specialties reported facing a direct question asking for a commitment. This is a direct and clear material breach of the MPA set forth by the NRMP. In addition, almost three-quarters of those who received such a question felt somewhat or very uncomfortable answering this question. Applicants also said the effect on their decision to rank a program soliciting a commitment was significant. Over 38% of applicants who received such a request were less likely to rank the program on their rank lists. This information is powerful for residency programs. Knowing that questions on commitment directly lower a program on the applicant’s rank list should encourage program directors to educate their interviewers that such questions are not only a direct violation but can significantly jeopardize recruiting efforts. Program directors could both improve compliance with the MPA and improve their recruiting and retention of highly qualified applicants by taking this information into account.

These results may be surprising to medical educators and program directors, but they are not to applicants. Medical student applicants share stories of commitment solicitation during the actual interview as well as post-interview communication between applicants and programs.^7^ Oftentimes, applicants feel a high degree of gamesmanship played on both sides of the residency application process. Anecdotally, medical students are contacted by programs to subtly, or not so subtly, gauge interest. In addition, programs who do not participate in post-match resident contact appear to be at a disadvantage. One program who explicitly chose not to participate in post-interview communication found on a post-match survey that if they had contacted applicants after interviews, the program may have done better in the match.^9^ While post-interview communication is important, program directors must be cognizant that compliance with the MPA can be beneficial to success in the match.

For program directors, a response might be one of clearly outlining to all interviewers what can and cannot be asked during a residency interview. It is imperative that residency programs have clearly stated policies about post-match contact. One such stance, which might limit the ‘gamesmanship,’ would be that such contact is ‘voluntary and has no bearing on an applicant’s standing.’ It is only once the process becomes more transparent that applicants’ anxiety and unethical behavior on the part of the residency programs can be lessened. The inherent mistrust on both sides has eroded the application process and created an atmosphere where code words and innuendos often lead to disappointment from both sides on Match Day.

## LIMITATIONS

This study shares limitations inherent in survey-based research projects. Completed potentially months after the interviews, the results were dependent on the memory of respondents. All responses were returned within 2 weeks of electronic mail and therefore, prior to match results being announced.

The response rate was 58.6%. The percentage of illegal interview experiences may be artificially increased as respondents who identified illegal or inappropriate questions may be more inclined to return the survey as a means of catharsis.

Survey results were based on the applicants’ interpretation of questions rather than actual questions asked. While there is no way to monitor all interviews for commitment questions, asking applicants immediately after the interview season offers a reasonable alternative. This would allow for the applicant to reflect on all of their interviews but may limit exact recall of specific questions.

In addition, recall bias may also play a role. An applicant may recall a request for commitment because they remember an uncomfortable interview better than a comfortable experience. This systematic bias might artificially increase the rate of these types of questions.

## CONCLUSION

Almost one in five respondents among the five major specialties surveyed were asked to commit to a program during the 2006–2007 residency interview season, often by multiple programs. This is an explicit violation of NRMP match rules. Program directors must realize such questions have a negative impact on their matched applicant list, a finding that may influence them to change their interview practices. Graduate medical education should consider educational outreach and information to both applicants and programs regarding acceptable interview procedures and guidelines. A formal “Interview Code of Conduct” might address a potentially flawed process for selecting future practitioners and medical educators.

## Figures and Tables

**Table 1:** Specialty distribution of commitment questions (N=912).

Answer	General surgery	Prelim surgery	Internal medicine	Prelim medicine	Emergency medicine	Obstetrics/gynecology	Orthopedic surgery	Total
Yes (%)	171 (22.53)	19 (19.00)	339 (16.78)	262 (23.04)	141 (15.86)	180 (24.10)	137(29.53%)	1,249
No (%)	588 (77.47)	81 (81.00)	1,681 (83.22)	875 (76.96)	748 (84.14)	567 (75.90)	327 (70.47)	4,867
Total	759	100	2,020	1,137	889	747	464	6,116

*N*, total number of responses

**Table 2:** Applicant comfort level of sharing information.

	Frequency	Percent
Very uncomfortable	424	30.72%
Somewhat uncomfortable	606	43.91%
Neither comfortable or uncomfortable	156	11.30%
Somewhat comfortable	119	8.62%
Very comfortable	62	4.49%
No response	13	0.94%

**Table 3:** Applicant effect on ranking.

	Frequency	Percent
Much more likely to rank it highly	38	2.75%
More likely to rank it highly	148	10.72%
No effect	647	46.88%
Less likely to rank it highly	388	28.12%
Much less likely to rank it highly	142	10.29%
No response	17	1.23%

**Table 4:** Specialty choice by comfort level.

	Comfort level of sharing information					
	
Specialty	Very uncomfortable	Somewhat uncomfortable	Neither comfortable or uncomfortable	Somewhat comfortable	Very comfortable	Total
Surgery (%)	53 (28.19)	80 (42.55)	26 (13.83)	17 (9.04)	12 (6.38)	188
Medicine (%)	178 (29.82)	267 (44.72)	76 (12.73)	53 (8.88)	23 (3.85)	597
Emergency medicine (%)	46 (32.86)	59 (42.14)	15 (10.71)	16 (11.43)	4 (2.86)	140
OBGYN (%)	55 (30.73)	87 (48.60)	13 (7.26)	16 (8.94)	8 (4.47)	179
Orthopedic surgery (%)	44 (32.59)	58 (42.96)	15 (11.11)	10 (7.41)	8 (5.93)	135
Total	376	551	145	112	55	1,239

	Affect on decision to rank the program					
	
	Much more likely to rank it highly	More likely to rank it highly	No effect	Less likely to rank it highly	Much less likely to rank it highly	Total
Surgery (%)	8 (4.23)	29 (15.34)	90 (47.62)	45 (23.81)	17 (8.99)	189
Medicine (%)	15 (2.53)	54 (9.09)	291 (48.99)	171 (28.79)	63 (10.61)	594
Emergency medicine	4 (2.88)	12 (8.63)	61 (43.88)	45 (32.37)	17 (12.23)	139
OBGYN (%)	4 (2.23)	18 (10.06)	81 (45.25)	59 (32.96)	17 (9.50)	179
Orthopedic surgery (%)	4 (2.99)	21 (15.67)	64 (47.76)	31 (23.13)	14 (10.45)	134
Total	35	134	587	351	128	1,235

*OBGYN*, obstetrics and gynecology

N = 141 was the frequency missing.

*OBGYN*, obstetrics and gynecology

No Response = 145.
